# Constructive Appraisal of “Mental Health Symptom Screening Among Adult Emergency Department Patients: A Cross-Sectional Study”

**DOI:** 10.1016/j.acepjo.2026.100366

**Published:** 2026-04-03

**Authors:** Preeti Lamba

**Affiliations:** Dr. D.Y. Patil Vidyapeeth (Deemed to be University), Pune, Maharashtra, India

To the Editor:

I read with interest the recent cross-sectional study evaluating the prevalence of anxiety, depression, and posttraumatic stress disorder (PTSD) symptoms among adult emergency department (ED) patients.^1^ The authors reported that more than half of the participants screened positive for at least 1 elevated mental health domain, with substantial symptom overlap across conditions. These findings provided important contemporary insight into the psychological symptom burden present within general ED populations.

The study is strengthened by its randomized bed-based recruitment strategy and use of validated screening instruments. By enrolling patients irrespective of presenting complaint, the investigators extend beyond crisis-centered samples and offer a broader assessment of behavioral health vulnerability in routine emergency care. The reported association between symptom burden and socioeconomic disadvantage aligns with recent evidence suggesting that EDs increasingly serve as access points for individuals with unmet mental health and social needs.^2^

Several considerations merit further discussion.

First, screening positivity should not be interpreted as diagnostic equivalence. Although the Patient-Reported Outcomes Measurement Information System and Primary Care PTSD Screen for Diagnostic and Statistical Manual of Mental Disorders, Fifth Edition, are validated tools for identifying elevated symptoms, the ED environment itself may contribute to transient distress. Acute illness, pain, uncertainty, and prolonged waiting periods may influence self-reported symptom severity. Contemporary research emphasizes that symptom screening in acute care settings may capture both enduring psychopathology and context-driven distress, underscoring the importance of cautious interpretation.^3^ Without confirmatory diagnostic evaluation or longitudinal follow-up, prevalence estimates may reflect a mixture of persistent and situational symptoms.

Second, the cross-sectional design limits inference regarding downstream clinical impact. Although quantifying symptom burden is essential, the practical value of systematic ED screening ultimately depends on whether identification leads to meaningful referral, engagement in outpatient care, or improved patient-centered outcomes. Implementation studies in emergency settings increasingly highlight that screening alone is insufficient unless paired with structured referral pathways and accessible follow-up services.^4^ Future research incorporating postdischarge outcomes would strengthen the translational relevance of these findings.

Third, the enrollment rate raises the possibility of selection bias. Individuals who consent to complete mental health surveys during an ED visit may differ from those who decline participation, potentially influencing prevalence estimates. Greater characterization of nonparticipants, where feasible, would help contextualize representativeness and external validity.

Despite these limitations, the study underscores the substantial burden of self-reported psychological symptoms among adult ED patients. As emergency medicine continues to integrate behavioral health within routine care, careful attention to diagnostic specificity, feasibility, and longitudinal effectiveness will be essential to ensure that screening strategies translate into meaningful clinical benefit.

In conclusion, this investigation contributes valuable epidemiologic data to the evolving discourse on mental health screening in emergency settings. Refinement of screening thresholds and evaluation of sustainable linkage models will be important next steps in advancing behavioral health integration within emergency medicine.

## Funding and Support

By *JACEP Open* policy, all authors are required to disclose any and all commercial, financial, and other relationships in any way related to the subject of this article as per ICMJE conflict of interest guidelines (see www.icmje.org). The authors have stated that no such relationships exist.

## Conflict of Interest

All authors have affirmed they have no conflicts of interest to declare.
